# Self-administered acupressure for treating adult psychiatric patients with constipation: a randomized controlled trial

**DOI:** 10.1186/s13020-015-0064-7

**Published:** 2015-11-03

**Authors:** Wai Kit Wong, Wai Tong Chien, Wai Ming Lee

**Affiliations:** School of Nursing, The Hong Kong Polytechnic University, Hung Hom, Kowloon, Hong Kong; Forensic Community Psychiatric Nursing Team, Castle Peak Hospital, Tuen Mun, Hong Kong

## Abstract

**Background:**

Constipation has a high prevalence rate (>30 %) in psychiatric patients with psychotropic drugs. Common pharmacological and non-pharmacological interventions for constipation might have longer-term negative and adverse effects that would outweigh their short-term efficacy in symptom reduction. This randomized controlled trial aims to investigate the effect of self-administered acupressure for the management of constipation, in hospitalized psychiatric patients.

**Methods:**

Seventy-eight patients were recruited in matched pairs in terms of gender, age and laxative use from five acute psychiatric wards in Hong Kong. Each of these matched pairs of patients was randomly assigned to either a self-administered acupressure (n = 39) or a sham group (n = 39), using computer-generated random numbers. After baseline measurement, the intervention and sham group received the same training in self-administered acupressure and supervised practice once per day for 10 days, except light pressure on non-acupoints was taught to the sham group. The acupoints chosen for acupressure included *Zhongwan* (RN12), right and left *Tianshu* (ST25), right and left *Quchi* (LI11). Participants’ symptoms and quality of life regarding constipation were measured at baseline and immediately and 2 weeks after completion of the interventions with constipation assessment scale and patient assessment of constipation quality of life questionnaire, respectively.

**Results:**

After 2 weeks follow-up, participants who had received self-administered acupressure indicated significantly greater improvements in both symptom severity (*P* = 0.0003) and quality of life (*P* = 0.0004) when compared with the sham group.

**Conclusion:**

The psychiatric patients with constipation who practiced self-administered acupressure for 10 days improved their symptom severity and perceived quality of life immediately and 2 weeks after completion of the intervention in comparison with the sham group.

Trial registration: The trial was registered with the ClinicalTrials.gov (Reg. No: NCT02187640)

**Electronic supplementary material:**

The online version of this article (doi:10.1186/s13020-015-0064-7) contains supplementary material, which is available to authorized users.

## Background

Constipation is a common and chronic physical health problem worldwide. In Hong Kong and the United States, the prevalence of constipation in the general population is about 14.3 and 12.0–19.0 %, respectively [1, 2]. Untreated constipation can have severe consequences such as fecal impaction and bowel obstruction, which may lead to extended periods of hospitalization, or even premature mortality [3, 4]. Constipation contributes to 13.7 million days of restricted activities and 3.4 million days of bed disability each year in the United States and several other developed countries [5–8]. More than $725 million per year is spent on laxatives for management of constipation in the United States [9]; similar high costs related to this disorder have been reported in Hong Kong and many Western countries [1, 4].

More than one-third of psychiatric patients who take psychotropic drugs (e.g., antipsychotics and antidepressants) suffer from constipation [10]. The high rate of constipation among psychiatric patients may result not only from the side effects of psychotropic drugs (mainly the inhibitory effects of anticholinergics on acetylcholine) [11], but also from patients’ limited exercise, sedentary lifestyle, negative symptoms (e.g., avolition and social withdrawal), loss of energy, poor mental state, poor dietary practice, and insufficient fiber intake [12, 13]. Psychiatric patients with constipation may feel embarrassed about seeking medical consultation and health advice and/or lack the initiative to change their unhealthy lifestyle and behaviors, resulting in negative consequences to their daily lives and health [14].

The most common pharmacological and non-pharmacological interventions for constipation can produce short-term symptom relief. Although pharmacological therapies such as laxatives can stimulate bowel movement, these medications may produce many long-term side effects (e.g., metabolic disturbances and hepatotoxicity) that can far outweigh their therapeutic effects of symptom reduction [14]. Non-pharmacological interventions can be categorized as traumatic (e.g., acupuncture) or non-traumatic (e.g., auriculotherapy, reflexology, behavioral therapy, and abdominal massage) [15–17]. Recent evidence suggests that acupuncture is effective at relieving symptoms of constipation [18–20]. However, acupuncture is a traumatic intervention, which may limit its clinical application as an alternative treatment. Non-traumatic and complementary therapies, such as auriculotherapy, behavioral therapy, and reflexology, have been seldom used or found ineffective in the treatment of constipation [16, 21]. In one randomized controlled trial, abdominal massage therapy decreased the severity of constipation and increased bowel movement after 8 weeks, but this did not lead to a decrease in laxative intake [22].

A number of other non-pharmacological complementary therapies with self-administerable procedures have been recommended for managing constipation in people with chronic illness [23]. Acupressure is the application of pressure to specific acupoints on the body and is believed to open up the flow of *qi*, the energy that restores balance within the human body [24]. Acupressure has been shown to be effective in the management of acute and chronic diseases, such as breathlessness in patients with chronic obstructive pulmonary disease, nausea and vomiting in cancer patients undergoing chemotherapy, and pain in those with dysmenorrhea [25–27]. Acupressure can improve symptoms of constipation in people with stroke [28] and neurological disorder [29], as well as older people residing in care homes for the aged [30]. However, there is limited evidence of the effects of acupressure in treating medication- and/or lifestyle-induced constipation in hospitalized psychiatric patients.

A “stepped care” system has recently been proposed to enhance both the efficiency and effectiveness of treatment for chronic health problems. The stepped care approach to patient care comprises a sequence of treatment options offered to patients, ranging from low-intensity and less expensive, optimal interventions to more complex and intensive approaches, depending on what is deemed necessary and beneficial for individual patients [31]. In the stepped care system, the self-help approach to illness management is a treatment approach that offers user-friendly, convenient, and less expensive interventions. In addition to reducing treatment costs, self-help treatments or interventions may also enhance the self-care ability and self-efficacy of service users, especially those with chronic illnesses [32]. Chinese medicine practitioners have developed and validated standardized manuals and procedures for acupressure as a user-friendly way to guide self-application of the technique among adult psychiatric patients [33].

This randomized controlled trial aims to investigate the efficacy of self-administered acupressure in treating constipation in hospitalized psychiatric patients. Such patients often experience major psychological distress because of symptoms of constipation. The constipation assessment scale (CAS) is a universal assessment tool with good reliability and validity for a diverse patient population with constipation [5, 11, 19]. The CAS has demonstrated satisfactory internal consistency, good test–retest reliability, and significant contrasts between cancer patients with constipation and healthy controls [34]. In addition, severe constipation can result in reduced life satisfaction [11]. The symptom-specific patient assessment of constipation quality of life questionnaire (PAC-QoL) is commonly used to assess perceived quality of life in relation to the life impacts of constipation in hospitalized psychiatric patients [3, 11]. This scale has demonstrated good internal consistency, test–retest reliability, and construct validity in patients with chronic constipation [35]. The Chinese versions of the CAS and PAC-QoL (Additional file 1) used in this trial were validated and indicated satisfactory content validity, and satisfactory levels of equivalence with the original version, test–retest reliability, and internal consistency in a pilot study of 20 Chinese adult psychiatric patients with constipation [33].

## Methods

**Fig. 1:**
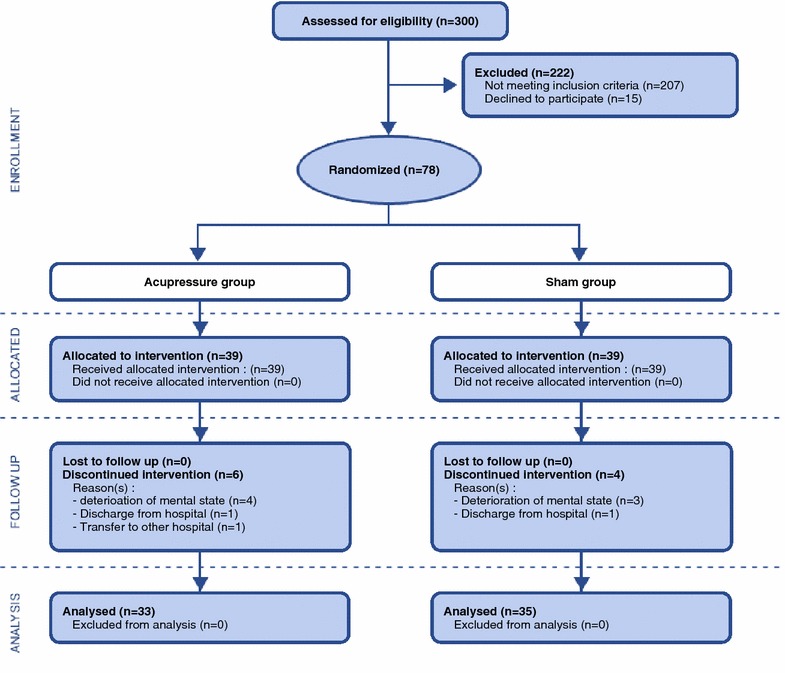
Recruitment, retention and group allocation of participants during the study

This was a randomized controlled trial with a repeated-measures sham-group design and was conducted between April and November 2013. A flow diagram of the study procedure is presented in Fig. 1, according to the CONSORT statement [36]. The controlled trial was registered with the ClinicalTrials.gov (Reg. No: NCT02187640).

In the absence of previous similar research on acupressure for constipation in psychiatric patients, the sample size calculation was based on Cohen’s standard medium effect size (*f* = 0.40 with 80 % power at 5 % alpha level) for healthcare and behavioral studies [37], taking into account an expected 20 % attrition rate [28–30]. It was calculated that 39 participants per group (78 participants in total) would be required for analysis of variance tests on the main study outcomes (symptom severity and quality of life), as calculated with G*Power version 3.13 (free download from MacUpdate at http://www.macupdate.com/apps). Seventy-eight eligible adult psychiatric patients volunteered to participate in this study. These patients were recruited over 6 months from five adult psychiatric wards in one 1000-bed regional mental hospital in Hong Kong. A few patients (n = 6) refused to participate, or were not selected because of lack of interest in participating or time constraints. Participants had been admitted to the hospital for acute psychiatric treatment (e.g., psychotropic medication) and rehabilitation before discharge.

Criteria for participation in this study included being: (1) Hong Kong Chinese residents, aged between 18 and 64; (2) willing and mentally competent to learn about, and engage in, self-care and acupressure, as assessed and recommended by the attending psychiatrists; and (3) diagnosed with constipation under the Rome III diagnostic criteria [38]. In relation to these criteria, participants must have presented in the past 6 months with the following symptoms of constipation: (a) rarely having loose stools without the use of laxatives, (b) insufficient evidence of irritable bowel syndrome, and (c) any two or more of the following cardinal symptoms:

1. Straining at defecation at least a quarter of the time,
2. Lumpy and/or hard stools at least a quarter of the time,
3. A sensation of incomplete evacuation at least a quarter of the time,
4. A sensation of anorectal obstruction/blockage at least a quarter of the time,
5. Manual maneuvers to facilitate defecation at least a quarter of the time, and/or,
6. Three or fewer defecations per week.

Participants with the following symptoms were excluded: (1) mental instability over the past month as indicated by their attending psychiatrist; (2) anatomical and physiological disorders of the gastrointestinal tract, such as mal-rotation, fistula, and colonic neuropathies; (3) various comorbidities of mental illness such as metabolic and endocrine diseases, lead poisoning, and vitamin D intoxication; (4) previous training in acupressure; and (5) physical disability involving the upper limbs or pregnancy.

The attending psychiatrist assessed and confirmed whether each participant was mentally competent to participate and whether they understood the instructions for the intervention and the responses to the outcome questionnaires. Informed written consent (Additional file 2) was obtained from participants judged competent to take part in the study. These participants were asked to complete baseline measures of socio-demographic characteristics and study outcomes. Participants were then matched in pairs according to gender, a factor associated with symptom severity and quality of life in previous studies on psychiatric patients with constipation [3, 14, 22]. Two sets of matched pairs of participants were listed and the paired participants in one list were then randomly assigned to either the self-administered acupressure or sham group using computer-generated random number sequences; their matched pairs were assigned to the corresponding study group. Participants in the sham group received similar training and implemented a similar acupressure procedure to the acupressure group, except that they were taught to use sham acupoints and perform abdominal massage with light pressure. Participants in this group were offered acupressure training and supervised practice after the study if the intervention was effective.

Training in self-administered acupressure for relieving constipation was performed according to a standard guideline or protocol for practice [39, 40]. This consisted of two simple procedures, as follows: first, the participant was taught to use the Acupen, a device used to apply constant pressure of 29.5–40.0 newton force onto five acupoints, lasting for approximately 1 min. The five acupoints were *Zhongwan* (RN12), which is located on the upper abdomen and on the anterior midline 4 cun (just over 10 cm) above the center of the umbilicus [29]; the right and left *Tianshu* (ST25), located in the central abdominal region and 2 cun (just over 5 cm) lateral to the umbilicus [39]; and the right and left *Quchi* (LI11), located at the lateral end of the transverse elbow crease when the forearm is flexed and at the midpoint of the line connecting *Chize* (LU5) to the lateral epicondyle of the humerus [41]. Participants were informed of the specific sensations (e.g., soreness and relaxation) that should be experienced when pressure is applied accurately to the acupoints. Second, gentle rubs (with the palm) with pressure on the abdomen in clockwise circular movements around 2 cun from the umbilicus were performed for another 8 min [42]. The positions of the acupoints and use of the Acupen were illustrated with colored photographs and pictures for the participants’ easy reference. The acupressure and abdominal massage were performed once a day for 10 days in a group setting, supervised by a trained psychiatric nurse and preferably at least 2 h after a meal. The main procedure and features of the acupressure intervention are summarized (in Additional file 3), according to the Standards for Reporting Interventions in Clinical Trials of Acupuncture (STRICTA).

The sham group was trained to perform acupressure in a similar way to the intervention group, except that they used five sham acupoints and mild abdominal massage with slight pressure only. Participants in the sham group were not told about the sensations (e.g., soreness and relaxation) induced by acupressure on the real acupoints. The acupressure and sham group programs were reviewed and agreed upon by a panel of six Chinese medical practitioners and acupressure therapists; minor amendments were made to a few terms about acupoints and the administration of acupressure in the programs.

A training workshop was conducted by an experienced Chinese medical practitioner who had practiced acupressure for 10 years. The workshop was designed to equip five mental health nurses (one in each psychiatric ward under study) with acupressure, abdominal massage, and group supervision skills. The trained nurses were then supervised to teach a group of patients about acupressure until the practitioner was satisfied with and confirmed their competence to train their patients in acupressure with the aid of a manual for self-administered acupressure. Each of the nurses also practiced training a group of four patients in self-administered acupressure under the supervision of the practitioner prior to the start of the study interventions. The treatment fidelity among the five trained nurses was confirmed by the research team according to the items in the treatment manual, and their performance was found to be 93–98 % accurate for all items.

### Instruments

A socio-demographic and clinical data sheet (Additional file 4) was completed at baseline. The primary and secondary outcome measures were scores on the Chinese versions of the CAS and the PAC-QoL questionnaire, respectively. These outcome measures were administered by the researcher (who was blind to participants’ group assignment) at recruitment, immediately after the 10-day interventions, and 2 weeks after the interventions.

The 8-item CAS has been frequently used to assess constipation symptoms in the previous 7 days, such as abdominal distention or bloating, frequency of bowel movements, oozing of liquid stool, and rectal pain with bowel movements. Subjects were asked to rate each item on a 3-point Likert scale: 0 = no problem, 1 = some problems, and 2 = severe problems. Higher total scores (possible range 0–16) indicate more severe constipation symptoms.

The 28-item PAC-QoL consists of four subscales: satisfaction with constipation symptoms and treatment (five items), physical discomfort (four items), psychosocial discomfort (eight items), and worries and concerns (11 items) [35]. Items are rated on a five-point Likert scale, ranging from 0 = none of the time or not at all to 4 = all of the time or extremely. The total and subscale scores were averaged by the total number of items, and then each average score (ranging from 0 to 4) was used for comparison. The lower the average total and subscale scores, the better the person’s perceived health-related quality of life.

### Data analysis

Descriptive statistics (frequency and mean [standard deviation]) were used to describe the socio-demographic data and scores on the outcome variables (PAC-QoL and CAS). The homogeneity of the two study groups was assessed by comparing their baseline outcome scores and socio-demographic characteristics using the Chi-square test (for nominal variables) and the independent sample *t* test or Mann–Whitney *U* test (for continuous variables). The mean scores of the two outcome variables were not normally distributed and there were significant differences in PAC-QoL mean scores between groups at the baseline. Therefore, the generalized estimating equation (GEE) test was used to compare differences in CAS and PAC-QoL mean scores between the treatment and sham groups across time (pretest and two post-tests), followed by contrast tests. For outcome measures showing significant results in the GEE test, subgroup analyses were performed to examine any significant differences in mean scores between subgroups for gender, age, type of antipsychotic drug used, use of laxatives, and tendency to suppress defecation. The level of significance for all statistical tests was set at 0.05.

Ethical approval (NTW-2012-0009) and permission to conduct the study were obtained from the Human Subjects Research Ethics Committee at The Hong Kong Polytechnic University and the Clinical Research Ethics Committee of the Hospital Authority, Hong Kong. The aim and procedure of the study were explained to the participants who met the study criteria and those who agreed to participate provided written consent.

## Results

### Characteristics of participants

Seventy-eight participants were recruited over 6 months and randomly allocated into either the self-administered acupressure or sham group (n = 39 in each group). Ten participants withdrew during the intervention period (attrition rate = 12.9 %) because of deterioration in their mental state; thus, 33 and 35 members of the acupressure and sham groups, respectively, completed the intervention and the two follow-up measures. Based on an examination of the participants’ diaries, the adherence rates of performing the acupressure therapy and sham program were 95.2 % (95 % CI 92.3–98.2 %) and 92.7 % (95 % CI 90.1–95.3 %), respectively, indicating non-significant differences between the groups.

The demographic and clinical characteristics of the 68 participants are summarized in Table 1. Most (n = 54, 79.4 %) had been diagnosed with schizophrenia (n = 29, 87.9 % and n = 25, 71.4 % in the acupressure and sham groups, respectively). Two-thirds (65.7 %) were aged between 41 and 60, and 33.3 % (n = 11) of the acupressure group and 37.1 % (n = 13) of the sham group were aged between 40 and 50. More than half (58.8 %; n = 19 in the acupressure and n = 21 in the sham group) of each group consumed less than 20–35 g/day of fiber (the World Health Organization recommends that 20–35 g/day as the optimal daily fiber intake [43]). Chi-square tests revealed no statistically significant differences between the treatment and sham groups on any of the demographic and clinical characteristics, indicating the homogeneity of the two groups at recruitment.

**Table 1:** Demographic and clinical characteristics of participants

	Total	Frequency	Treatment group	Frequency	Sham group	Frequency	Chi-square test	
	n = 68	%	n = 33	%	n = 35	%	Test value	*P*
Gender							0.108	0.743
Male	44	64.7	22	66.7	22	62.9		
Female	24	35.3	11	33.3	13	37.1		
Age							3.245	0.355
20–30	7	10.3	5	15.2	2	5.7		
30–40	15	22.1	5	15.2	10	28.6		
40–50	24	35.3	11	33.3	13	37.1		
50–60	22	32.4	12	36.4	10	28.6		
Diagnosis							5.020	0.170
Schizophrenia	54	79.4	29	87.9	25	71.4		
Bipolar disorder	1	1.5	1	3.0	0	0		
Depression	1	1.5	0	0	1	2.9		
Multiple diagnosis	12	17.6	3	9.1	9	25.7		
BMI							2.967	0.227
<18.5	3	4.4	1	3.0	2	5.7		
18.5–23.9	29	42.6	11	33.3	18	51.4		
>23.9	36	52.9	21	63.6	15	42.9		
Classification of occupation							0.675	0.714
Full-time	10	14.7	6	18.2	4	11.4		
Part-time	10	14.7	5	15.2	5	14.3		
Unemployment	48	70.6	22	66.7	26	74.3		
Highest education level							1.738	0.629
Primary	26	38.2	14	42.4	12	38.4		
Secondary	39	57.4	17	51.5	22	62.9		
Tertiary	2	2.9	1	3	1	2.9		
Not educated	1	1.5	1	3	0	0		
Living condition							0.021	0.870
Private/public housing	53	77.9	26	78.8	27	77.1		
Hostel	15	22.1	7	21.2	8	22.9		
Fluid intake							1.564	0.211
<1500 ml	30	44.1	12	36.4	18	51.4		
>1500 ml	38	55.9	21	63.6	17	48.6		
							0.041	0.839
Fiber intake								
<20–35 g/day	40	58.8	19	57.6	21	60		
>20–35 g/day	28	41.2	14	42.4	14	40		
Exercise more than twice per week							0.652	0.419
Yes	26	38.2	11	33.3	15	42.9		
No	42	61.8	22	66.7	20	57.1		
How long have you taken anti-psychotic drugs							1.244	0.265
<24 months	7	10.3	2	6.1	5	14.3		
>24 months	61	89.7	31	93.9	30	85.7		
Do you have constipation after taking anti-psychotic drugs							0.095	0.758
Yes	42	61.8	21	63.6	21	60		
No	26	38.2	12	36.4	14	40		
Do you taking laxatives or other drug for relieving constipation during study?							0.075	0.785
Yes	30	44.1	14	42.4	16	45.7		
No	38	55.9	19	57.6	19	54.3		
The total number of drugs used for relieving constipation							1.661	0.436
0	39	57.4	19	57.6	20	57.1		
1	16	23.5	6	18.2	10	28.6		
>1	13	19.1	8	24.2	5	14.3		
The total number of drugs used for treating psychiatric illness							0.231	0.891
1	17	25	9	27.3	8	22.9		
2–3	39	57.4	18	54.5	21	60		
>3	12	17.6	6	18.2	6	17.1		

### Mean scores of outcome measures at baseline

At the baseline measurement, there were no statistically significant differences in CAS mean scores (Mann–Whitney *U* = −0.614, *P* = 0.539) between the acupressure and sham groups. However, the PAC-QoL mean scores were significantly different between the two groups (Mann–Whitney *U* = −3.495, *P* < 0.0005); the acupressure group [0.87 (SD = 0.09)] had a significantly lower mean score on the PAC-QoL than the sham group [1.28 (SD = 0.09)]. Therefore, the baseline PAC-QoL mean scores were used as covariants for the analysis of the treatment effects.

### Treatment effects of self-administered acupressure

The mean scores on the CAS and the PAC-QoL (and its subscales) at recruitment, immediately after intervention, and 2 weeks after intervention, and comparisons between the two groups across time are summarized in Table 2. Over the 2-week follow-up, participants in the acupressure group showed significant improvements in CAS mean score and PAC-QoL mean score (Wald’s χ^2^ = 13.11 and 39.09, respectively, both *P* < 0.0005) compared with the sham group. There were also significantly greater improvements in the acupressure group in all domains of the PAC-QoL (physical, psychosocial, worries and concerns, and satisfaction) compared with the sham group (Wald’s χ^2^ = 17.70–39.09, all *P* < 0.0005). The mean scores for the CAS and PAC-QoL and its subscales for the pretest and two post-tests in the sham group were higher, indicating a worsening of both constipation symptoms and quality of life. The CAS mean score differences between the baseline and post-test 1 and between post-tests 1 and 2 were 0.18 and 0.94, respectively. The PAC-QoL mean score differences between the baseline and post-test 1 and between post-tests 1 and 2 were 0.08 and 0.28, respectively.

**Table 2:** Summary of outcome measures’ comparison between acupressure and sham groups before, immediate after intervention, and 2 weeks after intervention

	Treatment group (n = 33)			Sham group (n = 35)			GEE test		Effect size
	Pretest mean (SD)	Post-test 1 mean (SD)	Post-test 2 mean (SD)	Pretest mean (SD)	Post-test 1 mean (SD)	Post-test 2 mean (SD)	Wald’s χ^2^	*P*	
CAS	4.45 (0.404)	3.03(0.347)	2.64 (0.361)	4.94 (0.460)	5.12 (0.365)	5.88(0.487)	13.109	<0.0005	0.37
PAC-QoL									
Total	1.08 (0.712)	0.87 (0.728)	0.88 (0.719)	1.13 (0.695)	1.21 (0.686)	1.41 (0.679)	39.090	<0.0005	0.31
Physical	0.74 (0.105)	0.54 (0.087)	0.5 (0.103)	0.89 (0.114)	1.09 (0.098)	1.44 (0.121)	17.706	<0.0005	0.33
Psychosocial	0.41 (0.095)	0.30 (0.071)	0.27 (0.079)	0.96 (0.123)	0.87 (0.112)	1.03 (0.127)	21.513	<0.0005	0.30
Worries and concern	0.71 (0.091)	0.59 (0.088)	0.56 (0.089)	1.26 (0.107)	1.34 (0.111)	1.56 (0.120)	33.319	<0.0005	0.29
Satisfaction	2.05 (0.135)	1.79 (0.173)	1.76 (0.139)	2.37 (0.139)	2.63 (0.140)	2.78 (0.128)	17.694	<0.0005	0.32

*CAS* constipation assessment scale, *PAC-QoL* patient assessment constipation quality of life questionnaire, *SD* standard deviation, *GEE test* generalized equation estimation test

The results of contrast tests on the variables with significant treatment effects indicated that the participants in the acupressure group experienced significantly greater improvements in symptom severity at post-test 1 (mean difference = −2.00, *P* < 0.0005) and post-test 2 (mean difference = −3.14, *P* < 0.0005) than those in the sham group. There was a significant difference in quality of life between the groups at baseline. Changes in mean PAC-QoL scores from baseline to post-tests 1 and 2 were compared for the groups. The acupressure group reported a significantly greater reduction than the sham group in mean PAC-QoL scores at both post-test 1 (mean difference between groups = −0.36, *P* < 0.0005) and post-test 2 (mean difference between groups = −0.55, *P* < 0.0005). The CAS and PAC-QoL mean scores over the 2-week follow-up indicated that the self-help acupressure program demonstrated a significant, positive effect on constipation. In addition, the mean score differences in the acupressure group were significantly improved from the baseline measurement to post-test 1 and post-test 2 (mean difference = −1.42 and −1.81, respectively, for CAS and mean difference = −0.21 and −0.20, respectively, for PAC-QoL).

The subgroup analyses showed that those acupressure participants who had taken laxatives (n = 14) indicated significantly greater improvements in constipation symptoms (*P* < 0.0005) and perceived quality of life (*P* = 0.002) than those who had not used such medication over the 2-week follow-up. Despite these significant differences in treatment effects between the two subgroups, those acupressure participants who had not taken laxatives during the study period (n = 19) indicated significant within-group improvements in both constipation symptoms [i.e., the mean CAS score was reduced from 3.32 (SD = 0.279) at the baseline measurement to 2.32 (SD = 0.289) immediately after the intervention, and further reduced to 2.05 (SD = 0.361) at the 2-week follow-up] and perceived quality of life [i.e., the mean PAC-QoL score was reduced from 0.97 (SD = 0.072) at the baseline measurement to 0.94 (SD = 0.074) immediately after the intervention, even though it increased slightly to 1.02 (SD = 0.081) over the follow-up period].

## Discussion

There has been no controlled trial on the effects of self-administered acupressure in hospitalized psychiatric patients with constipation. In a study of older people with severe constipation, Lin et al. [30] used a similar treatment protocol (an acupressure program with abdominal massage) and a similar primary outcome (i.e., symptom severity). They reported a substantial effect on symptom reduction (effect size *f*^2^ = 3.03) immediately after the acupressure intervention. In the present trial, self-administered acupressure produced moderate effects in our sample of hospitalized adult psychiatric patients. The intervention not only reduced their symptom severity but also improved their perceived quality of life. These beneficial effects were sustained during the 2 weeks following trial completion. In this trial, both participants who took laxatives and those who did not demonstrate significant reduction of constipation symptoms and improvement of quality of life. The subgroup analysis showed that those who took laxatives experienced significantly greater symptom reduction than those who did not.

Acupressure and acupuncture enhance the functioning of visceral organs by activating the somatovisceral reflexes and modulating different biomechanical responses [44, 45]. In this controlled trial, all participants took at least one antipsychotic and/or mood stabilization drug with sedative and cholinergic side effects, including constipation. These sedative effects might slow down the functions of the autonomic system and counteract the positive effects of acupressure in relieving constipation. However, the self-administered acupressure and abdominal massage program in this trial demonstrated moderate overall treatment effect size (0.37, according to the GEE test) in reducing symptom severity in psychiatric patients with chronic constipation, particularly those with severe symptoms. However, CAS and PAC-QoL scores indicated some negative effects on constipation symptoms and deterioration of quality of life in the sham group over time. This might have resulted from ineffective treatment, side effects of the medications, or both; increased severity of constipation symptoms because of ineffective treatment would contribute to the reduction of these patients’ quality of life. However, the moderate effect produced by the 10-day acupressure program was also examined over a short-term (2-week) follow-up; the results suggested a sustained effect of acupressure treatment. The longer-term (e.g., >3 months) symptom-relieving effects of regular and consistent acupressure should be examined in diverse groups of psychiatric patients with constipation to explore differences related to age and symptom severity and chronicity. In addition, the self-help approach adopted for this acupressure program should be monitored and followed up over a longer period of time to ensure its sustainability and clinical efficacy in the treatment of constipation.

An assessment of health-related quality of life in patients with constipation or other chronic diseases can demonstrate how the illness or its symptoms affect patients’ well-being and daily activities of living [46]. In this trial, participants in the acupressure group reported significantly greater improvement in their perceived quality of life over the 2-week follow-up than participants in the sham group. The severity of patients’ constipation symptoms was negatively correlated with their perceived quality of life [47]. Using acupressure intervention to alleviate these disturbing symptoms, patients can re-establish their family and social activities, as well as their habits and hobbies, thus enhancing their life satisfaction and well-being [7, 46].

The self-help approach could save time and money for people with less complex or life-threatening health problems, who may otherwise (or unnecessarily) undergo more rigorous treatment procedures [23]. Efficacy in the self-management of an illness, and even other life situations, may also be enhanced if patients realize that they themselves, rather than a therapist, are responsible for their health condition [48]. Self-help programs may reduce the stigma of consulting and/or working with a therapist, particularly for those with severe mental illness [23, 49, 50]. The empowerment or promotion of self-help for patients with chronic illness could result in reduced negative feelings and enhanced efficacy in illness self-management, and subsequently lead to improved health-related quality of life [51].

Psychiatric patients could be trained and supervised to practice this self-administered acupressure program in small groups. These patients could then self-administer the intervention at any convenient time and place, such as their home and workplace, as preferred. As acupressure is a user-friendly treatment, most patients with chronic illness who are sufficiently physically fit to apply pressure to acupoints could easily adopt and implement this intervention with a simple treatment protocol to relieve their suffering from acute or chronic constipation.

It would be useful to conduct further research on the self-help approach to acupressure adopted in this trial over a longer-term follow-up. This would allow the investigation of whether efficacy in illness self-management can be enhanced in a range of patients with chronic constipation.

### Limitations of the study

First, this 14-day follow-up interval in this trial was based on previous controlled trials of acupressure for older people with constipation, which indicated significant effects on symptom alleviation over 1 week after completion of the intervention. Further research on the treatment effects of this self-administered acupressure beyond the 14-day follow-up (e.g., for 3–6 months) on a variety of clinical outcomes, such as family and social relationships, and even its cost-effectiveness, is recommended. Second, the intervention provided was time-limited to 10 days. Booster sessions could be added on an intermittent or regular basis to strengthen or ensure the persistent longer-term effects of acupressure. Service users’ preference for and satisfaction with the acupressure treatment regime should also be considered in future research. Third, this study adopted a convenience sample recruited from one single center, which might reduce the aptness of generalization and extrapolation from its findings. A multi-center controlled trial with a randomly selected sample of adult psychiatric patients is recommended to increase the validity and generalizability of the findings. Fourth, the patients in the sham group might have known that they were performing acupressure on the sham acupoints because there were no specific sensations (e.g., feelings of soreness) after applying pressure on each of the acupoints. Fifth, the trained nurse therapists of the sham group knew that the sham acupressure would be ineffective; this might have introduced subjective bias to the therapists and influenced their training performance with the sham group participants and consequently the treatment effects. Lastly, the study adopted a selective sample in which only mentally stable adult psychiatric in-patients were referred by their psychiatrists to participate. The application of this self-administered acupressure program to other patients with different types, severity and chronicity of mental illness, as well as diverse socio-demographic backgrounds, should be considered.

## Conclusion

The psychiatric patients with constipation who practiced 10 days of self-administered acupressure improved their symptom severity and perceived quality of life immediately and 2 weeks after completion of the intervention in comparison with the sham group.

## Additional files

10.1186/s13020-015-0064-7 Chinese versions of the CAS and PAC-QoL.
10.1186/s13020-015-0064-7 Written consent form.
10.1186/s13020-015-0064-7 Summary of the main procedure and features of the acupressure intervention.
10.1186/s13020-015-0064-7 Socio-demographic and clinical data sheet.

## Abbreviations

US: United States
CONSORT: Consolidated standard of reporting trials
ANOVA: Analysis of variance
CAS: Constipation assessment scale
PAC-QoL: Patient assessment constipation quality of life
GEE: Generalized estimating equation test
HRQoL: Health-related quality of life
