# Supplementary material for: Self-administered acupressure for treating adult psychiatric patients with constipation: a randomized controlled trial
Source: Chin Med. 2015 Nov 3;10:32. doi: 10.1186/s13020-015-0064-7 (PMC4630845; doi:10.1186/s13020-015-0064-7)
Supplement: Supplementary file 1 — 10.1186/s13020-015-0064-7 Chinese versions of the CAS and PAC-QoL. [file 13020_2015_64_MOESM1_ESM.pdf]

Chinese version of CAS

便秘評估量表(CAS)

請對以下每一題目圈出最適合你在最近七天內的情況:

沒有這種情況 , 有這種情況但不是很嚴重, 有這種情況但是很嚴重.

	項目	沒有這種情況	有這種情況但不是很嚴重	有這種情況但是很嚴重
**1**	腹脹 **Abdominal distension or bloating**	**0**	**1**	**2**
**2**	放屁量(次數)的改變 **Change in amount of gas passed rectally**	**0**	**1**	**2**
**3**	排便次數減少 **Less frequent bowel movements**	**0**	**1**	**2**
**4**	有流質大便從肛門滲出來<br><br>**Oozing liquid stool**	**0**	**1**	**2**
**5**	便意感(想大便的感覺) **Rectal fullness or pressure**	**0**	**1**	**2**
**6**	排便造成肛門內部(直腸)疼痛 **Rectal pain with bowel movement**	**0**	**1**	**2**
**7**	糞便的形狀變細<br><br>**Small stool size**	**0**	**1**	**2**
**8**	一直想要大便卻無法排出<br><br>**Urge but inability to pass stool**	**0**	**1**	**2**

總分 : ______________________

Chinese Verson of PAC-QoL

便秘症狀—病人自我評估

以下問題問及**過去兩星期內**便秘對您日常生活的影響。每條問題請 “✓”一格。

**以下問題問及過去兩星期內您症狀的嚴重程度。**	**沒有**<br>**0**	**少許**<br>**1**	**有些**<br>**2**	**很多**<br>**3**	**極多**<br>**4**
1. 您感到腹脹得快要破裂嗎?	☐	☐	☐	☐	☐
2. 您因便秘而覺得身體好像重了嗎?	☐	☐	☐	☐	☐

**以下幾條問題問及過去兩星期內便秘對您日常生活的影響。**	**沒有**<br>**0**	**偶然有**<br>**1**	**有時有**<br>**2**	**經常有**<br>**3**	**不停有**<br>**4**
3. 您感到身體不適嗎?	☐	☐	☐	☐	☐
4. 您感到需要大便但又不能排出來嗎?	☐	☐	☐	☐	☐
5. 您與別人一起時感到尷尬嗎?	☐	☐	☐	☐	☐
6. 您因不能排便而越吃越少嗎?	☐	☐	☐	☐	☐

以下幾條問題問及過去兩星期內便秘對您<u>日常生活</u>的影響。	沒有<br>0	少許<br>1	有些<br>2	很多<br>3	極多<br>4
7. 您需要特別小心飲食嗎?	☐	☐	☐	☐	☐
8. 您胃口有變差嗎?	☐	☐	☐	☐	☐
9 您有沒有因不能選擇食物而擔心 (例如在朋友家中)?	☐	☐	☐	☐	☐
10. 在家以外的其他環境中，您有沒有因要留在廁所太長時間而尷尬?	☐	☐	☐	☐	☐
11. 在家以外的其他環境中，您有沒有因太經常要去廁所而尷尬?	☐	☐	☐	☐	☐
12. 您有沒有因要改變日常生活規律而擔心(例如旅行、離家外出)?	☐	☐	☐	☐	☐
**以下幾條問題問及過去兩星期內您的<u>感受</u>。**	**沒有<br>0**	**偶然有<br>1**	**有時有<br>2**	**經常有<br>3**	**不停有<br>4**
13. 您因自己的狀況而感到容易不耐煩嗎?	☐	☐	☐	☐	☐
14. 您因自己的狀況而感到不開心嗎?	☐	☐	☐	☐	☐
15. 您因自己的狀況而使您不斷想著便秘的問題嗎?	☐	☐	☐	☐	☐
16. 您因自己的狀況而感到緊張嗎?	☐	☐	☐	☐	☐
17. 您因自己的狀況而感到自信心減少嗎?	☐	☐	☐	☐	☐
18. 您感到可以控制自己便秘的情況嗎?	☐	☐	☐	☐	☐

以下問題問及過去兩星期內您的<u>感受</u>。	沒有<br>0	少許<br>1	有些<br>2	很多<br>3	極多<br>4
19. 您有沒有因不知道何時才能排便而擔心?	☐	☐	☐	☐	☐
20. 您有沒有因需要排便但又不能排出而擔心?	☐	☐	☐	☐	☐
21. 您有沒有因不能排便而越來越困擾?	☐	☐	☐	☐	☐

以下問題問及過去兩星期內您有<u>便秘的生活</u>。	沒有<br>0	偶然有<br>1	有時有<br>2	經常有<br>3	不停有<br>4
22. 您害怕自己的狀況會惡化嗎?	☐	☐	☐	☐	☐
23. 您感到自己的身體運作得不正常嗎?	☐	☐	☐	☐	☐
24. 您大便的次數比您想要的少嗎?	☐	☐	☐	☐	☐

以下問題問及過去兩星期內您的<u>滿意程度</u>。	不滿意<br>0	少許滿意<br>1	有些滿意<br>2	很滿意<br>3	極滿意<br>4
25. 您滿意您的排便次數嗎?	☐	☐	☐	☐	☐
26. 您滿意您的排便規律嗎?	☐	☐	☐	☐	☐
27. 您滿意您的排便功能嗎?	☐	☐	☐	☐	☐
28. 您滿意您的治療嗎?	☐	☐	☐	☐	☐
