# Supplementary material for: Self-administered acupressure for treating adult psychiatric patients with constipation: a randomized controlled trial
Source: Chin Med. 2015 Nov 3;10:32. doi: 10.1186/s13020-015-0064-7 (PMC4630845; doi:10.1186/s13020-015-0064-7)
Supplement: Supplementary file 2 — 10.1186/s13020-015-0064-7 Written consent form. [file 13020_2015_64_MOESM2_ESM.pdf]

INFORMATION SHEET

Effect of self-administered acupressure on adult psychiatric in-patients with constipation

You are invited to participate in a join study conducted by School of Nursing, the Hong Kong Polytechnic University and Castle Peak Hospital. The aim of this study is to evaluate the effect of acupressure on adult psychiatric in-patients with constipation.

To understand the effect of self-administered acupressure on adult psychiatric in-patients with constipation, each participant will be participated in baseline measurement in terms of quality of life and constipation symptoms. The voluntary participants will be randomly assigned to two groups with the use of a computer generator the acupressure and sham group. All the participants will receive training in application of acupressure on relieving constipation (i.e., acupressure group learn acupressure protocol and sham group learn sham acupressure protocol), and perform self-administered acupressure under supervision until their competency to perform it by them. Then both groups performed intervention once a day for 10 days. Each session last about thirty minutes. For sham group participants, acupressure protocol will be taught to them if this intervention was effective. Two other outcome measurements will be conducted immediately after intervention and at 2-week follow up.

The modality of intervention in this study will not cause any injury or induce unnecessary pain. It is expected that this study will provide information and data for better understanding of the participants on the impact to quality of life, and effectiveness of acupressure on relieving constipation symptoms on adult psychiatric in-patients.

The participants should not undergo any additional intervention to manage constipation during the study period to avoid obscuring the real effects of the intervention.

You have entirely right to refuse to participate or withdraw from the study at anytime throughout the study. Your decision will not cause any detrimental effect to your normal intervention or assume any accountability to this research. All data collected from you will be kept confidential and for this study purpose only. Information is coded and coding is restricted to be accessed by the investigator only.

If you have any complaints about the conduct of this research study, please do not hesitate to contact Secretary of the Human Subjects Ethics Sub-Committee of The Hong Kong Polytechnic University in Person or in writing (c/o Human Resources Office in Room M1303 of the University).

If you would like more information about this study, please contact Mr. Wai Kit WONG or Mr. Wai Ming LEE at telephone number 2766 6774 and 2456 8512, respectively.

Thank you for your interest in participating in this study.

CONSENT TO PARTICIPATE IN RESEARCH

Effect of self-administered acupressure on adult psychiatric in-patients with constipation

I ____________________________ hereby consent to participate in the captioned research conducted by Mr. Wai Kit WONG & Mr. Wai Ming LEE.

I understand that information obtained from this research may be used in future research and published. However, my right to privacy will be retained, i.e., my personal details will not be revealed.

The procedure as set out in the attached information sheet has been fully explained. I understand the benefit and risks involved. My participation in the project is voluntary.

I acknowledge that I have the right to question any part of the procedure and can withdraw at anytime without penalty of any kind.

Name of participant ____________________________

Signature of participant ____________________________

Name of researcher ____________________________

Signature of researcher ____________________________

Date ____________________________
