# Supplementary material for: Self-administered acupressure for treating adult psychiatric patients with constipation: a randomized controlled trial
Source: Chin Med. 2015 Nov 3;10:32. doi: 10.1186/s13020-015-0064-7 (PMC4630845; doi:10.1186/s13020-015-0064-7)
Supplement: Supplementary file 3 — 10.1186/s13020-015-0064-7 Summary of the main procedure and features of the acupressure intervention. [file 13020_2015_64_MOESM3_ESM.pdf]

Table 1 Summary of main procedure and content of the acupressure intervention

Item	Detail	Page number
1. Acupuncture rationale	1)Traditional Chinese Medicine Acupressure was used and based on literature sources, and consensus methods	6, 11
2. Details of needling	2a) 5 acupoints were used per subject per session	10
	2b) Zhongwan (RN12), the right and left Tianshu (ST25), and right and left Quchi (LI11)	10
	2c) Apply constant pressure 3-5 kgf force with ‘Acupen’ onto each acupoint	9
	2d) The participants were informed of the specific sensations ‘de qi’ (e.g., soreness and flaccid) when accurately applying the pressure onto the acupoints.	10
	2e) Manual stimulation	9
	2f) Apply constant pressure onto each acupoint lasted about one minute	10
3. Treatment regimen	3a) 10 treatment sessions	10
	3b) Once per day and 13 minutes per each session	10
4. Other components of treatment	4a) Abdominal massage	10
	4b) Gentle rubs (use palm) with pressure on the abdomen in clockwise circular movements around 2 ‘cuns’ from umbilicus were performed	10
5. Practitioner background	5) A training workshop was run by an experienced traditional Chinese medical practitioner who had practiced acupressure for 10 years to equip five mental health nurses (one in each psychiatric ward under study) with acupressure, abdominal massage and group supervision skills. The trained nurses were then supervised to teach a few patients for acupressure in group until the practitioner satisfied with and confirmed their competence to train their patients in acupressure with an aid of a manual for self-administered acupressure. Each of the five trained nurses had also practiced training a group of four patients to learn the self-administered acupressure under the supervision of the practitioner prior to the start of the interventions in this controlled trial. The treatment fidelity among the five trained nurses was checked according to the items of the treatment manual by the research team and found to be 93-98% of accurate performance of all items.	11

6. Control or comparator interventions	6a) Sham control arms, like the placebo control arm in other controlled clinical trials, have the potential benefit of reducing the introduction of bias, particularly with regard to three critical areas of experimental design and conduct: treatment allocation, treatment adherence, and the assessment of subjective outcomes modified by treatment.	
	6b) The sham group was trained and performed acupressure in a very similar way as the intervention group, except the five sham acupoints and mild abdominal massage with slight pressure used.	10
6.1. Sham Acupuncture rationale	6.1)Traditional Chinese Medicine Acupressure was used and based on consensus methods	11
6.2. Details of needling	2a) 5 Sham acupoints were used per subject per session	10
	2b) Researchers developed non-acupoints was used as a placebo control in this study. The non-acupoints (A) which located on both elbows' lateral Epicondyle of the humerus and in the central abdominal region, 6 cun laterals to the umbilicus (B) and in the central abdominal region, 3 cun lateral to the umbilicus (C)	
	2c) Apply light pressure 1 kgf force with 'Acupen' onto each acupoint	10
	2d) The participants were informed of the no specific sensations 'de qi' (e.g., soreness and flaccid) when accurately applying the pressure onto the acupoints.	10
	2e) Manual stimulation	10
	2f) Apply constant pressure onto each acupoint lasted about one minute	10
6.3. Treatment regimen	6.3a) 10 treatment sessions	10
	6.3b) Once per day and 13 minutes per each session	10
