# Supplementary material for: Self-administered acupressure for treating adult psychiatric patients with constipation: a randomized controlled trial
Source: Chin Med. 2015 Nov 3;10:32. doi: 10.1186/s13020-015-0064-7 (PMC4630845; doi:10.1186/s13020-015-0064-7)
Supplement: Supplementary file 4 — 10.1186/s13020-015-0064-7 Socio-demographic and clinical data sheet. [file 13020_2015_64_MOESM4_ESM.pdf]

Demographic Data Sheet

1 **Personal data**

**1.1Diagnosis**	☐Schizophrenia[1]:_________________________________<br>☐Depression[2]:___________________________________<br>☐Bipolar Disorder[3]:_______________________________<br>☐Psychotic Disorder[4]:_____________________________<br>☐Others[5]:_______________________________________
**1.2Gender**	☐Male[1] ☐Female[2]
**1.3 Age**	☐18-24[1] ☐25-34[2] ☐35-44[3] ☐45-54[4] ☐≥55[5]
**1.4 BMI**	☐< 18[1] ☐18.5-22.9[2] ☐23-24.9[3] ☐25-29.9[4] ☐≥30[5]
**1.5 Classification of Occupation**	☐Full-time[1] ☐Part-time[2] ☐Unemployed[3]
**1.6 Highest Educational Level**	☐Primary[1] ☐Secondary[2] ☐Tertiary [3]☐Not educated[4]
**1.7Living condition**	☐Private[1] ☐Public[2] ☐ Hostel[3]
**1.8 Diet**<br>1.8.1 Fluid intake<br>1.8.2Fiber intake	 ☐<1500cc[1] ☐ ≥1500cc [2]<br>☐< 20-35g/day [1] ☐ ≥ 20-35g/day [2]
**1.10 Life style**<br>Exercise more than twice per week (each session more than half hour)	☐ Yes[1] ☐ No[2]

Clinical Data Sheet

2.1 **How long have you taken anti-psychotic drugs**<br><br>2.2 **Do you have constipation after taking anti-psychotic drugs**	☐< 24 months[1] ☐ ≥24 months[2]<br><br>☐ Yes[1] ☐ No[2]
2.3 **Do you taking laxatives or other drug for relieving constipation during study?**	☐ Yes[1] ☐ No[2]<br>(If Yes, Please answer2.4)
2.4.**Types of drug used for relieving constipation**	☐ Bulking agent [1] (Metamacil) ______________________________________<br>☐ Stimulant laxative [2] (Xanna, biscodyl) _____________________________<br>☐ Osmotic laxative [3] (Lactose) _____________________________________<br>☐ Stool softeners [4] (docusate sodium)_______________________________<br>☐ Others[5] ____
2.5 **The total number of drugs used for relieving constipation**	☐0[1] ☐1[2] ☐2[3] ☐>2[4]
2.6 **Types of drug used for treating psychiatric illness**	☐Antipsychotics(Conventional)[1] ___________________________________<br>☐ Antipsychotics(Atypical)[2] ___________________________________<br>☐Antidepressant[3] ___________________________________<br>☐Mood Stabilizers[4] ___________________________________

	☐Anticonvulants[5] ____________________________________<br>☐Anxiolytics[6] ____________________________________<br>☐Anti-parkinsonism[7] ____________________________________<br>☐Drugs Others[8]____
2.7 **The total number of drugs used for treating psychiatric illness**	☐0[1] ☐1[2] ☐2-3[3] ☐3-4[4] ☐>4[5]

Date of interview:____

Name of pariticpant:____

Name of researcher:____
